# Complete Genome Sequence of the Hypha-Colonizing *Rhizobium* sp. Strain 76, a Potential Biocontrol Agent

**DOI:** 10.1128/MRA.00571-20

**Published:** 2020-09-03

**Authors:** Rui-Liang Sun, Yu-Ling Jing, Rong-Jun Guo, Shi-Dong Li

**Affiliations:** a Institute of Plant Protection, Chinese Academy of Agricultural Sciences, Beijing, China; University of California, Riverside

## Abstract

The genome sequence of *Rhizobium* sp. strain 76, a bacterium isolated from the hyphosphere of Fusarium oxysporum f. sp. *cucumerinum*, is reported here. Genome sequencing and assembly yielded 5,375,961 bases with a 59.14% G+C content, comprising two chromosomes and one plasmid.

## ANNOUNCEMENT

Hyphosphere bacteria are closely related to host fungi and play important roles in plant health (1). *Rhizobium* sp. strain 76 was collected from the soil of a maize field in Langfang, Hebei, China, and isolated from the hyphosphere of the wilt-causing agent Fusarium oxysporum f. sp. *cucumerinum* (*Foc*) using a baiting method as described by Rudnick et al. (2). The strain was found to migrate along with *F. oxysporum* f. sp. *cucumerinum* hyphal growth and attenuated the disease incidence by 49% at a concentration of 10^8^ CFU ml^−1^. Here, we report the genome sequence of *Rhizobium* sp. strain 76 to reveal the genes related to biocontrol functions.

Strain 76 was assigned to the genus *Rhizobium* based on the 16S rRNA gene sequence (GenBank accession number MN719981) according to the method described by Gao et al. (3). For whole-genome sequencing, the DNA was extracted using the Wizard genomic DNA purification kit (Promega) after bacterial growth in Luria-Bertani broth (4) at 28°C for 10 to 12 h, and high-quality DNA (optical density at 260 nm [OD_260_]/OD_280_ ratio range, 1.8 to 2.0; >20 μg) was sequenced at Shanghai Meiji Biomedical Technology Company using two platforms. Briefly, Illumina sequencing libraries of 400- to 500-bp sheared fragments were prepared with the NEXTflex rapid DNA-Seq kit and used for paired-end sequencing (2 × 150 bp) on the HiSeq X Ten platform, while an ∼10-kb insert library for PacBio sequencing was sequenced on one single-molecule real-time (SMRT) cell following the standard protocol. The sequence data were analyzed using the free online bioinformatics platform Majorbio Cloud Platform. Low-quality data were removed based on the quality trimming statistics, and *de novo* assembly of paired-end reads was conducted using Canu 1.3 (5). The complete genome assembly consisting of seamless chromosomes and plasmids generated at the last circular step was manually checked, and the PacBio assembly was corrected with the Illumina sequencing data using Pilon 1.23 (6). The numbers of chromosomes and plasmids were determined by a BLASTN search of the reference sequences in the NCBI database and compared to the chromosome assignment of the published genome of *Rhizobium* sp. strain IRBG74 (NCBI assembly accession number GCA_000499645.1). The genome was annotated using the NCBI Prokaryotic Genome Annotation Pipeline (PGAP) (7).

A high-quality data set with a sequencing depth of 500× was generated. The *N*_50_ value of the PacBio raw reads was 14,808 bp, and 222,341 reads with an average length of 12,460 bp were generated. The Illumina sequencing generated 5,625,417 clean reads. The size of the whole genome is 5,375,961 bases, with two chromosomes and one plasmid. The average G+C content is 59.14%, and the genome contains 5,094 genes. The whole-genome analysis will help us to explore biocontrol mechanisms and facilitate the broad application of strain 76.

### Data availability.

This whole-genome shotgun project has been deposited at DDBJ/ENA/GenBank under the BioProject number PRJNA633024, BioSample number SAMN14930919, and the accession numbers CP053856 through CP053858. The raw Illumina and PacBio data have been deposited in the SRA database under the BioSample numbers SAMN14930919 and SAMN15069577 with the accession numbers SRR11794541 and SRR11887692, respectively.
